# Supplementary material for: MetaRibo-Seq measures translation in microbiomes
Source: Nat Commun. 2020 Jun 29;11:3268. doi: 10.1038/s41467-020-17081-z (PMC7324362; doi:10.1038/s41467-020-17081-z)
Supplement: Supplementary file 10 — Supplementary Data 7 [file 41467_2020_17081_MOESM10_ESM.zip › File2/Confidence_High_Taxonomy/132605_out.krona.html]

Javascript must be enabled to view this page.

members
magnitude
magnitudeUnassigned
count
unassigned
taxon
rank

132605\_out

181

superkingdom
2
181

181
976
phylum

181
200643
class

171549
order
181

171550
family
1

1
genus
239759

1
28117

SRS893170\_contig\_number\_2716
species

12
family
815

816
genus
12

3

SRS055966\_contig\_number\_63SRS063127\_contig\_number\_689SRS063985\_contig\_number\_22943
1638780
species

28116

SRS142542\_contig\_number\_18742
species
1

1
species

SRS144603\_contig\_number\_5241
2292941


SRS143876\_contig\_number\_31366
species
1262742
1

1

SRS148319\_contig\_number\_12911
species
29523

species

SRS017433\_contig\_number\_4612
310297
1

1
species

SRS143722\_contig\_number\_6019
46506

671267

SRS056519\_contig\_number\_619
species
1

1
47678

SRS050752\_contig\_number\_20444
species

species

SRS017247\_contig\_number\_3455
821
1

family
2005525
167

167
375288
genus

2292241

SRS1055099\_contig\_number\_7547
species
1

46503

SRS011302\_contig\_number\_19598SRS011405\_contig\_number\_12283SRS013687\_contig\_number\_31077SRS013800\_contig\_number\_10890SRS013940\_contig\_number\_3564SRS014235\_contig\_number\_17727SRS014415\_contig\_number\_23SRS014613\_contig\_number\_11890SRS014923\_contig\_number\_1935SRS014948\_contig\_number\_13613SRS015217\_contig\_number\_25530SRS015663\_contig\_number\_14889SRS015694\_contig\_number\_15081SRS015782\_contig\_number\_21681SRS016018\_contig\_number\_16188SRS016056\_contig\_number\_730SRS016132\_contig\_number\_182SRS016203\_contig\_number\_2170SRS016335\_contig\_number\_30916SRS016381\_contig\_number\_621SRS016840\_contig\_number\_143SRS017103\_contig\_number\_26584SRS017521\_contig\_number\_46657SRS017701\_contig\_number\_19055SRS017859\_contig\_number\_1762SRS018049\_contig\_number\_355SRS018133\_contig\_number\_13573SRS018888\_contig\_number\_1604SRS018936\_contig\_number\_3640SRS019178\_contig\_number\_799SRS019286\_contig\_number\_4954SRS019381\_contig\_number\_10691SRS019397\_contig\_number\_9425SRS019808\_contig\_number\_14637SRS020233\_contig\_number\_25314SRS020271\_contig\_number\_337SRS023526\_contig\_number\_21174SRS023914\_contig\_number\_25100SRS024075\_contig\_number\_1544SRS024331\_contig\_number\_22366SRS024435\_contig\_number\_37389SRS024492\_contig\_number\_20438SRS042628\_contig\_number\_22242SRS042966\_contig\_number\_10273SRS043667\_contig\_number\_4332SRS043841\_contig\_number\_8066SRS045004\_contig\_number\_9697SRS045244\_contig\_number\_3787SRS045645\_contig\_number\_29027SRS045713\_contig\_number\_2032SRS045826\_contig\_number\_22023SRS046068\_contig\_number\_128SRS047014\_contig\_number\_17976SRS047433\_contig\_number\_3721SRS048981\_contig\_number\_798SRS049402\_contig\_number\_14300SRS049712\_contig\_number\_21035SRS049773\_contig\_number\_17729SRS049896\_contig\_number\_19644SRS049959\_contig\_number\_28108SRS050299\_contig\_number\_12471SRS050422\_contig\_number\_30268SRS050520\_contig\_number\_322SRS050925\_contig\_number\_15446SRS051031\_contig\_number\_27395SRS051610\_contig\_number\_9244SRS051882\_contig\_number\_10559SRS052697\_contig\_number\_18354SRS053356\_contig\_number\_34069SRS053398\_contig\_number\_contig-100\_1753.120740SRS053649\_contig\_number\_19527SRS054352\_contig\_number\_contig-100\_2600.128017SRS057480\_contig\_number\_5681SRS058070\_contig\_number\_14368SRS062427\_contig\_number\_7301SRS062654\_contig\_number\_15469SRS063190\_contig\_number\_9394SRS063370\_contig\_number\_contig-100\_2080.59434SRS063489\_contig\_number\_1199SRS063813\_contig\_number\_contig-100\_16647.16648SRS064232\_contig\_number\_777SRS064276\_contig\_number\_29004SRS064757\_contig\_number\_20224SRS065176\_contig\_number\_9864SRS065397\_contig\_number\_3023SRS065504\_contig\_number\_17913SRS075021\_contig\_number\_879SRS075341\_contig\_number\_9766SRS075821\_contig\_number\_13661SRS076804\_contig\_number\_12568SRS076976\_contig\_number\_3331SRS077024\_contig\_number\_9282SRS077086\_contig\_number\_4848SRS077231\_contig\_number\_10903SRS077294\_contig\_number\_contig-100\_3806.97745SRS077454\_contig\_number\_contig-100\_18199.58395SRS077849\_contig\_number\_19295SRS078176\_contig\_number\_11890SRS078419\_contig\_number\_18725SRS078665\_contig\_number\_9659SRS097920\_contig\_number\_19679SRS097958\_contig\_number\_5668SRS098061\_contig\_number\_2273SRS098571\_contig\_number\_14490SRS098717\_contig\_number\_19308SRS101268\_contig\_number\_54SRS101376\_contig\_number\_1455SRS101433\_contig\_number\_7480SRS103987\_contig\_number\_28201SRS104036\_contig\_number\_4409SRS1041031\_contig\_number\_11116SRS1041039\_contig\_number\_169SRS1041091\_contig\_number\_12433SRS1041092\_contig\_number\_2303SRS1041112\_contig\_number\_2788SRS1041118\_contig\_number\_16983SRS1041130\_contig\_number\_1142SRS1041134\_contig\_number\_8735SRS1041137\_contig\_number\_contig-100\_1400.150707SRS1041138\_contig\_number\_13974SRS1041139\_contig\_number\_1487SRS1041140\_contig\_number\_12700SRS1041141\_contig\_number\_2101SRS104311\_contig\_number\_23521SRS104327\_contig\_number\_1178SRS104400\_contig\_number\_43193SRS104912\_contig\_number\_14522SRS104975\_contig\_number\_13285SRS105153\_contig\_number\_25532SRS1054716\_contig\_number\_8790SRS1055022\_contig\_number\_1217SRS1055069\_contig\_number\_5645SRS1055076\_contig\_number\_11499SRS1055095\_contig\_number\_contig-100\_97.98SRS142503\_contig\_number\_18388SRS142890\_contig\_number\_17662SRS143148\_contig\_number\_18940SRS143181\_contig\_number\_11246SRS144135\_contig\_number\_27509SRS144183\_contig\_number\_1108SRS144506\_contig\_number\_24071SRS144537\_contig\_number\_31581SRS145497\_contig\_number\_10712SRS146764\_contig\_number\_20163SRS146812\_contig\_number\_64227SRS146813\_contig\_number\_23276SRS147139\_contig\_number\_31318SRS147346\_contig\_number\_33269SRS147557\_contig\_number\_2013SRS147614\_contig\_number\_2836SRS148091\_contig\_number\_9348SRS148196\_contig\_number\_33845SRS148253\_contig\_number\_8588SRS148784\_contig\_number\_13778SRS148817\_contig\_number\_2962SRS149075\_contig\_number\_2312SRS149181\_contig\_number\_23332SRS893230\_contig\_number\_6655SRS893252\_contig\_number\_2960SRS893292\_contig\_number\_3319SRS893295\_contig\_number\_8777SRS893300\_contig\_number\_8116SRS893327\_contig\_number\_2421SRS893382\_contig\_number\_519SRS893383\_contig\_number\_26525
species
165

1

SRS1055043\_contig\_number\_24422
species
2290935

171551
family
1

1
1952616

SRS055982\_contig\_number\_11295
species
